# Estimating Interoceptive Sensitivity from Physiological Breathing Parameters

**DOI:** 10.1002/snz2.70050

**Published:** 2026-05-11

**Authors:** Ella McLeod, Faye Gorman, Bruce R. Russell, Phoebe Chin, Olivia K. Harrison

**Affiliations:** ^1^ Department of Psychology University of Otago Dunedin New Zealand; ^2^ Ngāpuhi New Zealand; ^3^ Ngāti Kahu New Zealand; ^4^ School of Pharmacy University of Otago Dunedin New Zealand; ^5^ Nuffield Department of Clinical Neurosciences University of Oxford Oxford UK; ^6^ Translational Neuromodeling Unit University of Zurich and ETH Zurich Zurich Switzerland

**Keywords:** breathing, inspiratory resistance, interoception, interoceptive measurement, sensitivity

## Abstract

Impairments in interoception (the process of sensing, perceiving, and interpreting internal stimuli from the body) are considered characteristic of many mental health conditions. Although there is a growing body of interoceptive research, accounting for the inherent physiological variability associated with interoceptive stimuli is a key challenge, particularly when estimating measures of sensitivity. Therefore, the purpose of this project was to develop and evaluate a physiological‐adjusted measure of breathing‐related interoceptive sensitivity. Seventy‐seven participants completed questionnaires exploring general affect, anxiety and self‐rated measures of interoception alongside a respiratory resistance sensitivity task. The proposed physiology‐adjusted sensitivity metric (perceptual threshold) was positively correlated with symptoms of maladaptive anxiety and depression. In comparison, the previously established counterpart (absolute intensity) was positively correlated with state anxiety and the Multidimensional Assessment of Interoceptive Awareness Questionnaire (MAIA) emotional awareness subscale. Comparisons using Steiger's Z Test indicated the MAIA not‐distracting subscale was more strongly correlated with the physiology‐adjusted metric than its standard counterpart. Thus, the proposed metric of interoceptive sensitivity provides a measure that can adequately account for physiological variability and relates to maladaptive anxiety and depression.

## 1 Introduction

Interoception is the process of perceiving and interpreting internal bodily sensations (Khalsa et al. 2018; Nord and Garfinkel 2022). Although much of our sensory processing occurs outside of conscious awareness (Connell et al. 2018; Stein and Peelen 2021), individuals may choose to ‘tune in’ or ‘cast awareness towards’ particular interoceptive channels, particularly when these are considered to hold valuable information regarding one's own safety. Alongside the role interoception plays in both homeostasis (an internal state of equilibrium) and allostasis (the process of maintaining homeostasis through change; Engelen et al. 2023; McEwen 2000), these perceptual processes also shape cognition, decision making, and emotional experiences (Tacca 2011; Tsakiris and Critchley 2016; Sugawara et al. 2020).

Despite increased interest in interoceptive research (Chen et al. 2021), several methodological challenges persist. These challenges have contributed to the conflicting results found both within and between interoception domains (Ehlers and Breuer 1992; Harrison, et al. 2021). Interoceptive signals (and their measurement) are inherently noisy, which makes the development of robust measures of specific interoceptive dimensions a significant challenge (Desmedt et al. 2023; Murphy 2024). Notably, measurement and analysis techniques should account for inter‐ and intra‐individual physiological differences within the interoceptive modality of choice, while also providing the granularity required to accurately identify and assess an individual's perceptual threshold and/or objective perceptual performance (Desmedt et al. 2023).

Breathing‐related interoception has more recently gained traction as a viable modality for measuring a subset of interoceptive properties (Paulus 2013; Garfinkel, et al. 2016; Harrison, et al. 2021; Nikolova et al. 2022). While interoceptive measures are not considered interchangeable across modalities (Garfinkel, et al. 2016), in comparison to cardiac tasks, breathing signals are typically readily available for conscious perception (Garfinkel, et al. 2016; Harrison, et al. 2021; Nikolova et al. 2022). Breathing is often consciously controllable, and breathing based stimuli can more easily be manipulated to adjust task performance for robust measures of interoceptive sensitivity (Garfinkel, et al. 2016; Harrison, et al. 2021; Nikolova et al. 2022). While breathing perceptions themselves are multifaceted and contain signals such as muscular tension and blood gas‐driven instincts to breathe (Banzett et al. 2008), one common methodological avenue to evoke breathing‐related interoceptive stimuli is by utilising different levels of inspiratory resistance (Paulus 2013; Garfinkel, et al. 2016; Harrison, et al. 2021; Nikolova et al. 2022). Tasks such as the filter detection task (FDT; Garfinkel, et al. 2016; Harrison, et al. 2021) and the respiratory resistance sensitivity task (RRST; Nikolova et al. 2022) were designed to apply a variable inspiratory resistance to facilitate measurement of breathing‐related interoceptive sensitivity. The level of sophistication when measuring breathing‐related perceptions of inspiratory resistances has rapidly and markedly improved in a short period of time (Nikolova et al. 2022). For example, the first use of the FDT required measurement of 20 trials at every level of resistance to find a participant's perceptual threshold (Garfinkel, et al. 2016), while the next iteration utilised a Bayesian model to hold task performance within a 65%–80% accuracy range (Harrison, et al. 2021; Nikolova et al. 2022).

More recently, the RRST has utilised a classic psychophysical technique, a psychometric function, to infer more intricate measures of interoceptive sensitivity such as the perceptual threshold (a measure of absolute sensitivity) and slope (a measure of relative sensitivity; Kingdom and Prins 2010; Harrison, et al. 2021; Nikolova et al. 2022). The psychometric function provides a way to quantify the relationship between an observer's task performance (for example, accurate perception), and a characteristic of a stimulus (for example, the intensity of an inspiratory resistance; Kingdom and Prins 2016). Importantly, this approach allows a perceptual threshold to be modelled from somewhat noisy subjective measures and provides added granularity above the discrete stimulus levels chosen within a task (Gold and Ding 2013; Kingdom and Prins 2016).

Previous research using the RRST has utilised the psychometric function to estimate perceptual sensitivity according to the resistance applied (i.e., via the percentage obstruction imposed by the apparatus to create inspiratory resistance; Nikolova et al. 2022). However, not only is the resistance generated at each obstruction position variable due to slight mechanical variability (such as stepper motor drift; Nikolova et al. 2022), participant‐driven differences in the inspiratory pressure generated against this resistance can vary from trial to trial via differences in breathing rate and depth. This can be seen by following an analogous application of Ohm's Law,

ΔP = R×Q,

where the change in inspiratory pressure (Δ*P*) is proportional to flow (*Q*) generated against a static inspiratory resistance (*R*) (Kaminsky 2012). Therefore, a trial where a participant takes a faster inhalation (that generates greater inspiratory flow) will result in a larger inspiratory pressure than a slower inhalation (with a smaller inspiratory flow; Kaminsky 2012; Nikolova et al. 2022) against the same resistance. A participant who consistently inhales sharply, compared to softly, throughout the task is likely to produce more perceptible stimuli (thus distinguishing between stimuli is easier), and would be considered to have “better” sensitivity at the same level of resistance intensity (Kingdom and Prins 2010; Kaminsky 2012). As such, the inclusion of physiological variability has been reported to be the critical next step in interoceptive methodological development in the breathing domain (Nikolova et al. 2022). Incorporating trial‐wise physiological parameters of inspiratory pressure will therefore allow us to more robustly quantify perceptual sensitivity towards detecting inspiratory resistances. Consequently, the aim of the current project was to develop an analytical protocol to incorporate physiological variability into interoceptive sensitivity metrics related to perceiving inspiratory resistances.

## 2 Methods

### 2.1 Participants and Recruitment

Data were collected from 77 participants (65 female, 11 male, one gender fluid), aged 18–42 years (M = 22.8, SD = 5.0), as part of two studies. Fourteen pilot participants were recruited for a study (Ethics Reference HDEC 20/CEN/168) exploring the effects of longitudinal interventions (including exercise and medications) on anxiety. Inclusion criteria required participants to be aged 18–45, exercise no more than once per week, and meet several health‐related criteria (e.g., no regular medication use, no chronic medical conditions, and no history of serious psychiatric disorders). Data for the remaining 63 participants was taken at baseline as part of an acute intervention study (exploring progressive muscle relaxation and breathing exercises; Ethics Committee Reference number H23/061). Inclusion criteria required participants: be aged 18–45; not be experiencing acute or chronic medical, neurological, and/or mental health disorders; not be currently taking medication; not have previous experience of the acute interventions; and reporting moderate levels of trait anxiety (defined as a State–Trait Anxiety Inventory (STAI)—Trait score of over 40; Spielberger et al. 1983).

Participants were recruited from the community via online advertisements, and participants for the acute intervention study were also recruited through the University of Otago SONA Research Participation System (for undergraduate students enrolled in 100‐level and 200‐level papers). A power analysis conducted using G^*^Power (version 3.1.9.6) indicated a sample size of 72 participants would be required to observe a moderate effect size (Cohen's *q* = 0.50) using Steiger's Z test for comparing two dependent correlations, with 80% power and a two‐tailed alpha of 5%.

### 2.2 Questionnaires

Participants completed a set of questionnaires designed to assess affective qualities and self‐reported interoceptive beliefs on a computer. Affective qualities were measured using the Generalised Anxiety Disorder 7 Item Scale (GAD‐7; Spitzer et al. 2006), the STAI (Spielberger et al. 1983), and the Centre for Epidemiologic Studies Depression Scale (CESD) (Radloff 1977). The Multidimensional Assessment of Interoceptive Awareness Questionnaire (MAIA); (Mehling et al. 2012) was used to measure self‐reported interoceptive beliefs.

### 2.3 Respiratory Resistance Sensitivity Task

**FIGURE 1:**
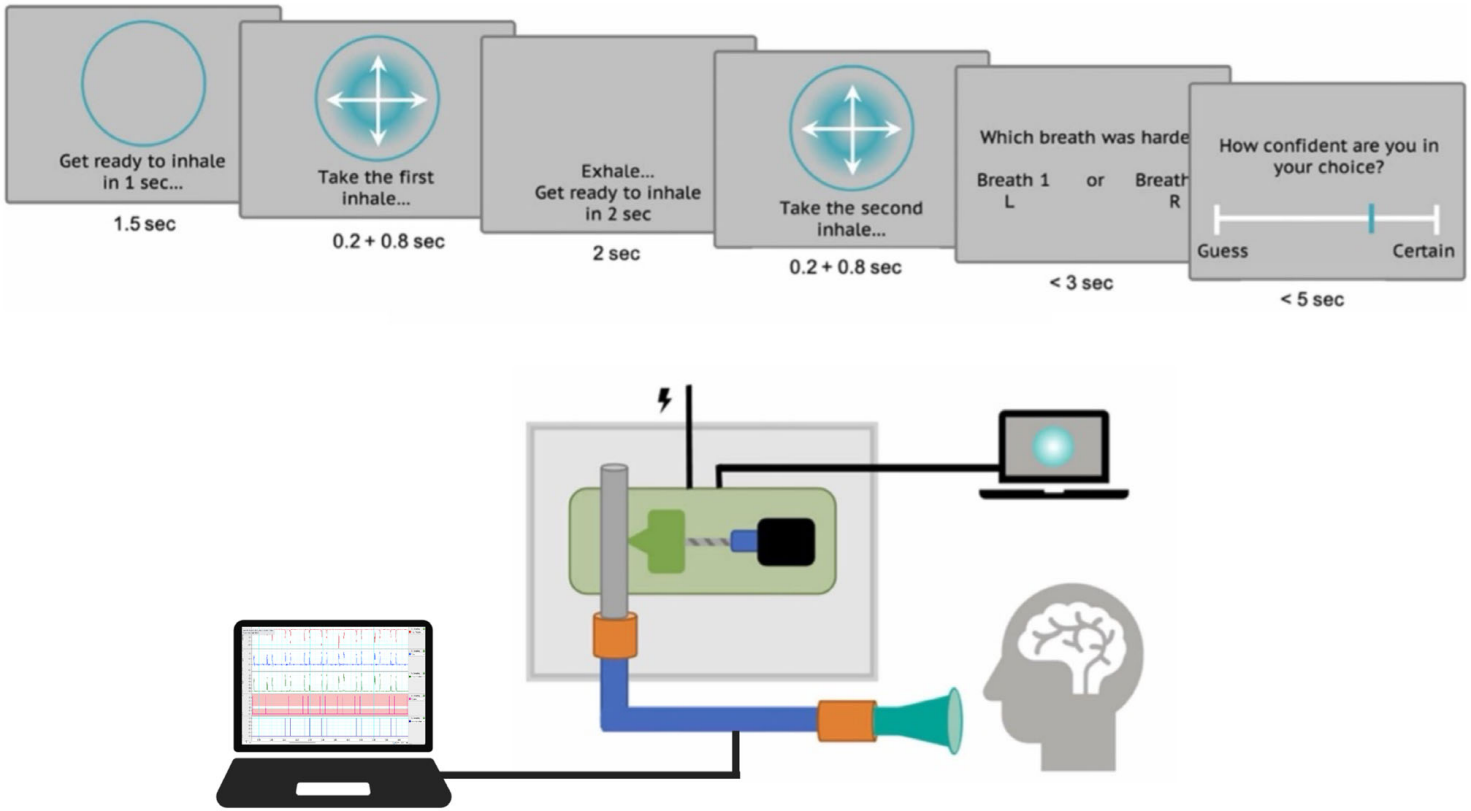
Slides depict visual prompts for the RRST. The task followed a two interval forced choice design, with the inclusion of a confidence rating to measure breathing‐related interoceptive confidence. The schematic depicts basic apparatus design. Figure is adapted from Nikolova et al. (2022). CC BY 4.0.

The RRST was used to obtain breathing‐related interoceptive perceptual data. To begin, participants were provided with written instructions for the task, which were then repeated verbally while they familiarised themselves with the RRST apparatus (Figure 1). Following familiarisation, the task was calibrated and administered by a computer programme. As seen in Figure 1, participants were cued to “prepare to breathe”, then altered their breath to synchronise with the expansion of a Gaussian ring. Participants were required to take two prompted breaths in each trial, then indicate which breath was more strongly resisted (using the left and right mouse buttons), and rate their confidence in their decision. Between each trial, participants were asked to remove the mouthpiece to minimise fatigue and data interference. Participants completed eight practice trials to become familiar with the pace of the task and the computer decision/rating interface. Following the practice trials, participants completed five blocks of 20 trials (100 trials and 200 breaths in total). Between each block of trials at least 30 s of rest were enforced to prevent hyperventilation.

#### 2.3.1 RRST Equipment

The RRST apparatus, as shown in Figure 1, uses a custom 3D printed device that applies resistance by constricting a piece of flexible tubing, effectively decreasing the diameter of the tubing. Participants breathed through a single use filtered mouthpiece (POWERbreathe, Southam, United Kingdom) connected to the RRST apparatus through a breathing circuit. Mechanical obstruction by the RRST apparatus upon the flexible tubing resulted in participants experiencing various degrees of restricted inspiration (with unrestricted expiration) when breathing through the mouthpiece. Physiological traces, specifically flow and pressure, were also measured throughout the task, extending the experimental setup initially proposed by Nikolova et al. (2022). Inspiratory flow and pressure were quantified using a spirometer (ADInstruments, Dunedin, New Zealand) and pressure transducer connected to a bridge amplifier (ADInstruments) within the breathing circuit, which was connected to a PowerLab (ADInstruments). These physiological parameters were recorded, alongside breath prompt timings, using LabChart 8 software (ADInstruments). Throughout the task, participants wore a nose clip to ensure they were only breathing through the RRST circuit, and listened to pink noise via headphones to obstruct environmental noise.

#### 2.3.2 RRST Computer Programme and Adaptive Procedure

Alongside guiding breaths, the RRST programme implemented an adaptive staircase procedure (Psi) to dynamically modulate the stimulus intensity (by adjusting the mechanical obstruction of the apparatus) such that a participant's perceptual threshold could be determined (Nikolova et al. 2022). Psi is a Bayesian adaptive psychophysical method that predicts the threshold and slope of the psychometric function based on response accuracy to prior trials (Kontsevich and Tyler 1999). Psi dynamically adjusts the stimulus intensity for each trial to maximise information gain regarding the parameters of the psychometric function (Kontsevich and Tyler 1999). Code to run the RRST task and instructions for creating the apparatus can be found in the original publication (Nikolova et al. 2022).

### 2.4 Data Processing

Three participants were excluded from the analyses due to errors in the physiological data files, and a further 14 participants were excluded as task performance was below 70%. Performance below 70% was associated with poor physiological signal quality and inconsistent respiratory behaviour, resulting in insufficient data for accurate psychometric estimation. Consequently, the final sample consisted of 60 participants with complete RRST and questionnaire data. Questionnaires were scored according to their respective published guidelines. An additional general interoceptive belief score was derived from the average score from six of the MAIA subscales (Noticing, Attention Regulation, Emotional Awareness, Self‐Regulation, Body Listening, and Trusting; Vig et al. 2022; Rominger and Schwerdtfeger 2024). The Not‐Worrying and Not‐Distracting scales demonstrate limited association with the MAIA g‐factor and were thus excluded from the general interoceptive belief score (Vig et al. 2022; Rominger and Schwerdtfeger 2024).

### 2.5 Physiological Data Processing

Physiological data from the RRST were recorded and preprocessed using dedicated physiological recording software (LabChart 8; ADInstruments), and MATLAB (24.1.0.2837808 (R2024a) Update 7). Peak inspiratory pressure and flow for each prompted breath (indicated by automatic trigger recordings from the RRST) were extracted using custom‐written MATLAB scripts.

#### 2.5.1 Interoceptive Sensitivity Measures

Interoceptive sensitivity was quantified using two measures. The original measure, absolute intensity, represents the percentage of obstruction at the perceptual threshold. This value was calculated using the α (alpha) parameter of the psychometric function estimated using Psi, in which the stimulus levels corresponded to incremental obstruction steps. As the detectable difference in inspiratory resistance is influenced by both the degree of obstruction and trial‐to‐trial physiological differences in the pressure and flow generated against the obstruction, a measure of breathing‐related interoceptive sensitivity that can adequately account for intra‐ and inter‐individual physiological variability is required (Kaminsky 2012; Nikolova et al. 2022). The proposed measure, a physiology‐adjusted perceptual threshold, is derived from the α (alpha) parameter of a psychometric function in which the stimulus levels correspond to relative physiological stimulus intensity calculated using a Weber Contrast (Haigh et al. 2021).

### 2.6 Physiology‐Adjusted Perceptual Threshold

To create a measure of breathing‐related sensitivity which more accurately accounts for physiological variability, the physiological stimulus intensity at each trial was calculated using Weber's Contrast. This approach is adapted from Weber's Law, which states the ratio of just‐noticeable difference to the baseline stimulus intensity remains constant (Haigh et al. 2021, Sowden 2012). While this law is not universal across all sensory modalities or intensities, the resulting Weber's Contrast is a standard measure for quantifying stimulus sensitivity (Sowden 2012). Weber's Contrast,

$$\text{Δ}P\;=\;\frac{\text{Δ}S}{S}\;,$$

was applied to the peak inspiratory pressure value of each prompted breath, where the change in stimulus intensity (Δ*P*) is determined by the ratio of stimulus change (Δ*S;* difference in peak inspiratory pressure between breaths) to the baseline stimulus intensity (*S;* the pressure of the unresisted breath; Haigh et al. 2021). In this context Δ*P* represents the computed contrast‐based intensity measure for a specific trial (Δ*S/S*).

Outlier trials for the difference in peak pressure were identified using the scale estimator Sn, as proposed by Rousseeuw and Croux (1993). The use of the scale estimator Sn is particularly suited for data with unknown and potentially skewed distributions, such as those encountered in the measurement of perceptual dimensions. The Sn values were computed using the RousseeuwCrouxSn() function (Jones 2019) in MATLAB, applied to the physiological stimulus intensities (Δ*P*) calculated using the Weber Contrast. Outliers were defined as those physiological stimulus intensities where the median distance exceeded three times the scale estimator (Jones 2019, Paire et al. 2023).

The remaining physiological stimulus intensities were subsequently used as an input parameter (*x*) in a psychometric function to model the perceptual threshold for each individual, while accounting for physiological variability. The Weibull psychometric function was used to obtain an estimate of the threshold (α) for each participant:

$$\psi \left(\chi ;\alpha ,\beta ,\gamma ,\lambda \right)=\gamma +\left(1-\gamma -\lambda \right)\;\times \;1-e^{-{\left(x/\alpha \right)}^{\beta }},$$

where *ψ* represents the proportion of correct responses at stimulus intensity, *χ*. Gamma (*γ*) is the guess rate and *λ* is the lapse rate. Threshold, *α*, the stimulus level which corresponds to a specified response probability, and *β*, the gradient of the function at the designated threshold. The Weibull psychometric function was deemed most appropriate as (physiological) stimulus intensities increased on a linear scale, and when stimulus intensity was equal to zero there was no difference between the stimuli (Kingdom and Prins 2010). While the slope parameter (*β*) is often used as a relative measure of sensitivity, it was not employed for this purpose in our project as we had insufficient within‐participant data for accurate estimation: While a minimum number of trials has not been established for accurate estimation of slope, the suggestion is 400 trials (Kingdom and Prins 2010). An appropriate value at which to fix slope (*β* parameter) was therefore estimated using a grand mean psychometric function. The grand mean Weibull psychometric function was fit across all participants, using the Bayesian Criterion with the PAL_PFBA command from the Palamedes toolbox in MATLAB (Prins and Kingdom 2018). Fixed values were used for the guess (0.5) and lapse (0.03) rates (Kingdom and Prins 2010; Prins 2019) to avoid overspecification of the model. The search grid was defined using the same parameters as in Nikolova et al. (2022); threshold uniformly spaced within the range [1.9476, 18.2091], based on the 10th to 99.99th percentile of the inverse psychometric function; slope uniform log(1) to log(16)). To confirm adequacy of the specified parameter space (search grid), posterior distributions were assessed using contour plots. Posterior contour plots indicated adequate parameter identifiability, with no multimodality, significant correlations, or convergence concerns. Finally, individual Weibull psychometric function fits were then estimated using PAL_PFML_Fit with a maximum likelihood criterion from the Palamedes toolbox in MATLAB (Prins and Kingdom 2018), where the estimated group *β* parameter (from the Bayesian fit grand mean psychometric function) was used as the fixed *β* value. Individual maximum likelihood estimation model fits were assessed using deviance and associated *p*‐value, as calculated using PAL_PFML_GoodnessOfFit (Palamedes toolbox; Prins and Kingdom 2018).

The proposed measure for interoceptive sensitivity was quantified as the physiology‐adjusted perceptual threshold (α parameter) from the Weibull psychometric function fit to individual participants using maximum likelihood estimation. The perceptual threshold represents the point at which a participant can perceive differences in inspiratory pressure (induced by breathing against a resistance at a trial‐specific inspiratory flow rate) at an above chance level (conventionally recognised as 75% accuracy; Kontsevich and Tyler 1999). Lower threshold values indicated higher breathing‐related perceptual sensitivity, as participants were able to detect very slight inspiratory resistance loads.

### 2.7 Statistical Analysis

Statistical analyses were performed using R (R version 4.2.2, Rstudio version 2025.05.0 + 496). To examine the relationships between affective and perceptual variables, Spearman rank correlations were conducted using the psych:corr.test function in R. False Discovery Rate correction was applied to the variables of interest (absolute intensity and physiology‐adjusted threshold) to control for multiple comparisons (Benjamini and Hochberg 1995). Differences in the correlations between the absolute intensity and physiology‐adjusted threshold with all other variables were tested using Steiger's Test (Steiger 1980). For each outcome, a Z‐statistic and associated *p*‐value were calculated.

## 3 Results

### 3.1 Psychometric Function Fit

**FIGURE 2:**
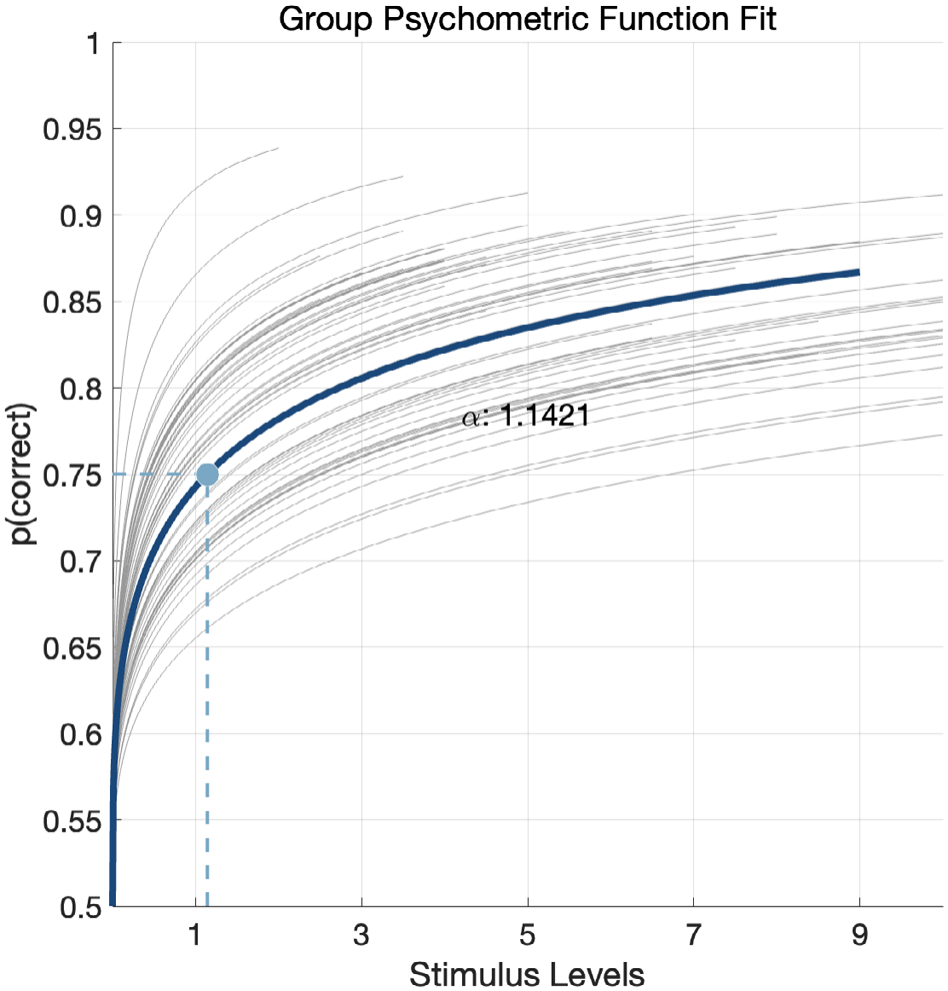
The Grand mean Bayesian psychometric fit (dark blue line) overlaid on individual psychometric maximum likelihood psychometric fits (grey lines), demonstrating the ability to quantify individual perceptual performance within this framework. The stimulus level which corresponds to 75% probability of a correct response is indicated by the dashed lines, and α gives the Bayesian grand mean estimated value.

The group psychometric function fit (Figure 2) illustrates the relationship between the stimulus intensity (trial‐wise difference in Weibull contrast pressure values) and the probability of a correct response across all participants. The fit was estimated using a Bayesian psychometric modelling approach, with the group threshold defined as the stimulus level at which participants were predicted to achieve 75% accuracy. From the Bayesian grand mean fit, the threshold (α) and slope (*β*) parameters are estimated as 1.14 and 0.34, respectively. Individual psychometric function fits were then assessed, with poor fit defined as deviance greater than 30, and/or a *X* ^2^ *p*‐value < 0.05 (Kingdom and Prins 2010). Poor fit was identified for nine participants (5.4%); these participants were retained in the primary analysis to preserve statistical power and a sensitivity analysis without their inclusion was conducted to assess the influence of these participants.

Descriptive statistics for individual‐level perceptual variables are reported in Table 1. Mean accuracy was 78.05% (SD = 0.02), indicating the RRST algorithm successfully held participant performance at an above chance level. The median absolute intensity (percentage obstruction) was 85.80% (IQR = 81.25% to 89.23%). The median physiology‐adjusted threshold (α) estimate (from individual psychometric functions fit using maximum likelihood) was 1.94 (IQR = 1.02 to 4.53).

**TABLE 1:** Perceptual results.

Interoceptive metric	Median score	Lowerquantile	Upper quantile
Sensitivity (Physiology‐adjusted perceptual threshold)	1.94	1.02	4.53
Sensitivity (Absolute intensity; Percentage obstruction)	85.80	81.25	89.23
Accuracy (%)	78.00	77.00	79.00

### 3.2 Questionnaire Results

Median scores and interquartile range for each questionnaire and subscale are presented in Table 2. Participants reported moderate symptoms of anxiety and depression. The median GAD‐7 score of 8.00 (IQR = 4.75 to 11.00) is indicative of mild to moderate maladaptive anxiety, and the STAI median state (45.00, IQR = 38.75 to 47.00) and trait (50.00, IQR = 41 to 54.25) scores are indicative of high state and high trait anxiety, respectively (Spielberger et al. 1983; Spitzer et al. 2006). The median CESD score, 19.50 (IQR = 15.00 to 25.25) indicating severe symptoms of depression. Participants self‐reported interoceptive beliefs (as measured by the MAIA subscales) were within the standard deviation of those reported by (Mehling et al. 2018), suggesting self‐reported interoceptive belief is similar to the global population average.

**TABLE 2:** Questionnaire results.

Questionnaire	Median score	Lower quantile	Upper quantile
Depression (CESD)	19.50	15.00	25.25
Maladaptive Anxiety (GAD7)	8.00	4.75	11.00
MAIA Total Score (General Interoceptive Belief Score)	2.50	2.12	3.02
MAIA Attention Regulation	2.36	1.96	3.00
MAIA Body Listening	1.83	0.92	2.67
MAIA Emotional Awareness	3.20	2.40	3.85
MAIA Not Distracting	2.00	1.33	2.67
MAIA Noticing	3.00	2.00	3.42
MAIA Not Worrying	2.50	1.75	3.25
MAIA Self‐Regulation	2.50	1.44	3.00
MAIA Trusting	3.00	2.33	4.00
STAI State	45.00	38.75	47.00
STAI Trait	50.00	41.00	54.25

### 3.3 Correlation Results

**FIGURE 3:**
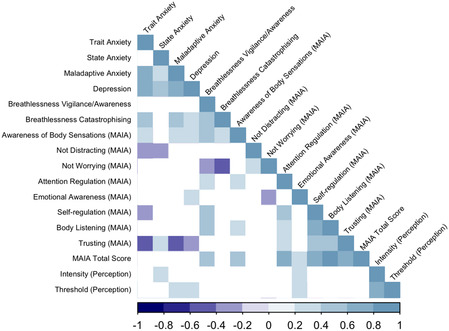
Correlation matrix containing the Spearman's correlation coefficients for questionnaire scores and interoceptive variables. Results are uncorrected for multiple comparisons. Only statistically significant (*p* < 0.05, uncorrected) correlations are visualised. Intensity (perception), absolute intensity; threshold (perception), physiology‐adjusted threshold.

The correlation matrix, Figure 3, illustrates statistically significant correlations (*p* < 0.05 uncorrected) between all variables. Of specific note, absolute intensity was positively correlated with state anxiety (Spearman's rho = 0.32, *p* = 0.01) and the emotional awareness MAIA subscale (rho = 0.33, *p* = 0.009). In contrast, the physiology‐adjusted threshold was positively correlated with maladaptive anxiety, as measured by the GAD‐7 (rho = 0.32, *p* = 0.01), depression as measured by the CESD (rho = 0.31, *p* = 0.02) and the emotional awareness MAIA subscale (rho = 0.28, *p* = 0.03). Following FDR correction for multiple comparisons applied to absolute intensity and physiology‐adjusted threshold correlations, absolute intensity was positively correlated with state anxiety (rho = 0.32, FDR corrected *p* = 0.06) and the emotional awareness MAIA subscale (rho = 0.33, FDR corrected *p* = 0.06) at a trend‐level, and physiology‐adjusted threshold was positively correlated with anxiety (rho = 0.32, FDR corrected *p* = 0.09) and depression (rho = 0.31, FDR corrected *p* = 0.09) at trend‐level.

**FIGURE 4:**
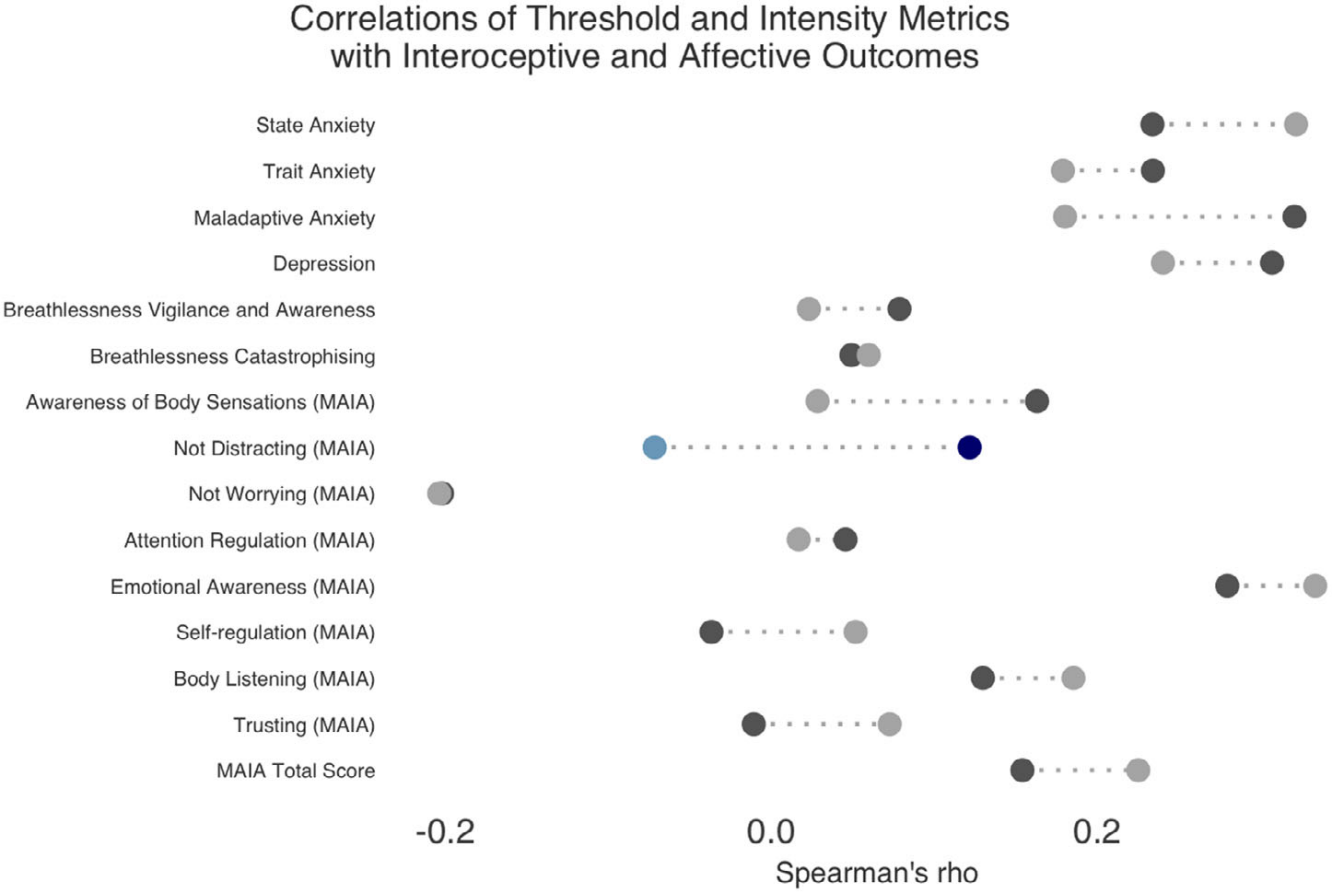
Comparison of Spearman's correlation coefficients between physiology‐adjusted threshold and absolute intensity with different affective and perceptual variables. Significant differences in correlation are illustrated in colour (*p* < 0.05, FDR corrected). Physiology‐adjusted threshold is indicated by the darker colour (dark blue and dark grey), absolute intensity is the lighter blue (where the difference is significant) and light grey (where the difference is not significant).

To compare the correlations between absolute intensity and physiology‐adjusted threshold with the affective and perceptual variables, pairwise r‐to‐z comparisons were conducted using Steiger's Z test. As shown in Figure 4, statistically significant differences in correlation were observed for the MAIA not‐distracting subscale. Higher scores on the MAIA not‐distracting subscale are usually considered to be adaptive and suggest an individual is tuned in to unpleasant bodily sensations. The MAIA not‐distracting subscale was more strongly correlated with Threshold (rho = 0.12) than with absolute intensity (rho = −0.07), *Z* = 2.16, *p* = 0.03.

## 4 Sensitivity Analysis

**FIGURE A1:**
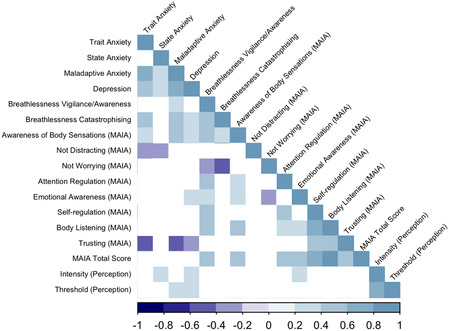
Sensitivity analysis correlation matrix. Correlation matrix containing the Spearman correlation coefficients for questionnaire scores and interoceptive variables. Results are uncorrected for multiple comparisons. Only statistically significant (*p* < 70.05, uncorrected) correlations are visualised. Intensity (perception), absolute intensity; threshold (perception), physiology‐adjusted threshold.

**FIGURE A2:**
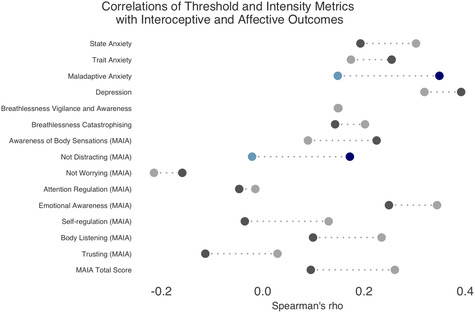
Comparison of Spearman correlation coefficients. Comparison between physiology‐adjusted threshold and absolute intensity with different affective and perceptual variables. Significant differences in correlation are illustrated in colour (*p* < 0.05, FDR corrected). Physiology‐adjusted threshold is indicated by the darker colour (dark blue and dark grey), absolute intensity is the lighter blue (where the difference is significant) and light grey (where the difference is not significant).

A sensitivity analysis (associated Figures A1 and A2, in the Appendix) was conducted after excluding nine participants with poorly fit individual psychometric functions. Following FDR correction for multiple comparisons, absolute intensity was no longer significantly correlated with state anxiety (*p* = 0.12, FDR corrected) or the MAIA emotional awareness subscale (*p* = 0.11, FDR corrected). Physiology‐adjusted perceptual threshold remained positively correlated with depression symptoms (*p* = 0.04, FDR corrected) and maladaptive anxiety (GAD‐7) at a trend level (*p* = 0.06, FDR corrected), however, it was no longer significantly correlated with the MAIA emotional awareness subscale (*p* = 0.25, FDR corrected). Additionally, maladaptive anxiety (GAD‐7) was more strongly correlated with physiology‐adjusted threshold (rho = 0.35) than with absolute intensity (rho = 0.15), *Z* = 2.37, *p* = 0.02. The MAIA not‐distracting subscale also remained more strongly correlated to threshold (rho = 0.17) than intensity (rho = −0.02), *Z* = 2.20, *p* = 0.03.

## 5 Discussion

The purpose of this project was to develop and evaluate a physiology‐adjusted measure of breathing‐related interoceptive sensitivity. In the proposed approach, trial‐wise differences in pressure serve as stimulus levels in a psychometric function from which the perceptual threshold is estimated to quantify interoceptive sensitivity. This physiology‐adjusted approach accounts for intra‐ and inter‐individual physiological variability, and compared to the standard RRST sensitivity metric (absolute intensity, which quantifies sensitivity based on the percentage obstruction at the perceptual threshold), it produces a measure of interoceptive breathing‐related sensitivity that better relates to trait levels of anxiety and subjective interoceptive measures.

Here we found that the standard RRST sensitivity metric, absolute intensity, was positively correlated with state anxiety (STAI State anxiety). As such, participants who expressed higher state anxiety had lower interoceptive breathing‐related sensitivity (greater absolute intensity), and this correlation remained following FDR correction (*p* < 0.1), but not following the sensitivity analysis that excluded participants with poorly fitting psychometric functions. However, state levels of anxiety are likely to influence respiratory patterns, thus, increasing physiological variability (Paulus 2013), which is not accounted for with the standard sensitivity metric of absolute intensity (percentage obstruction). Observed differences in interoceptive sensitivity with this metric may therefore reflect state‐dependent physiological fluctuations rather than true differences in the underlying perceptual sensitivity.

In contrast, our proposed physiology‐adjusted measure was instead positively correlated with elevated trait symptoms of maladaptive anxiety and depression. These correlations remained following FDR correction and following the sensitivity analysis at trend level (*p* < 0.1). Therefore, greater symptoms of maladaptive anxiety and depression appear to be associated with lower interoceptive breathing‐related sensitivity when using a physiology‐adjusted metric. This relationship did not extend to state anxiety (or trait anxiety measured using the STAI; Spielberger et al. 1983), which may suggest lower breathing‐related sensitivity is specifically associated with maladaptive instances of anxiety and depression.

Our findings are in line with previous research which suggests anxiety is related to dysfunctional breathing‐related interoception (Paulus 2013; Garfinkel, et al. 2016; Harrison, et al. 2021). However, in contrast, recent research by Banellis et al. (2025) has found breathing‐related interoceptive ability, including sensitivity as measured by the standard RRST sensitivity metric (absolute intensity), is unrelated to mental health symptoms. This highlights the importance of using a sensitivity metric that can account for physiological variance when working with populations with recognised intra‐ and inter‐individual variance. Additionally, close to 20% of the sample from Banellis et al. (2025) exhibited clinically significant levels of trait anxiety and close to 10% of the sample exhibited moderate to severe symptoms of depression, whereas a far greater proportion of our sample exhibited clinically significant levels of trait anxiety and depression (over 70% of the sample). This may suggest dysfunctional interoceptive processes are more prevalent among those with severe mental health symptoms.

The MAIA emotional awareness subscale, which measures an individual's awareness of the connection between body sensations and emotional states, was positively correlated with both absolute intensity and physiology‐adjusted threshold (only the correlation between absolute intensity and emotional awareness remained following FDR correction, at trend level, *p* < 0.1; Eggart and Valdés‐Stauber 2021). A higher score on the emotional awareness subscale indicates greater self‐reported interoceptive belief, which is therefore related to worsened objective sensitivity measures. This may indicate dissonance between objective interoceptive ability and subjective interoceptive beliefs, which is increasingly recognised as a factor for many mental and physical health disorders (Palser et al. 2018; Koreki et al. 2020). In contrast to our findings, Banellis et al. (2025) found the MAIA emotional awareness subscale was unrelated to interoceptive breathing related sensitivity. However, a greater proportion of our sample exhibited clinically significant anxiety and depression, which are commonly negatively correlated with the MAIA emotional awareness subscale (Banellis et al. 2025, Dunne et al. 2021).

The MAIA not‐distracting subscale, which measures an individual's tendency to not ignore or distract oneself from unpleasant body sensations, was more strongly correlated with physiology‐adjusted threshold (positive correlation) than absolute intensity (negative correlation), in the pairwise r‐to‐z comparison. Higher scores on this MAIA subscale suggest an individual has better ability to “tune in” to unpleasant bodily sensations, whereas low scores suggest a tendency to distract oneself or ignore unpleasant sensations (Eggart et al. 2021). As noted above, alongside the positive correlation between the physiology‐adjusted threshold and the MAIA not‐distracting subscale, the physiology‐adjusted threshold was positively correlated with maladaptive anxiety and depression. This suggests that individuals with maladaptive anxiety and depression may believe they are highly attuned to unpleasant bodily sensations (higher self‐reported interoception) but have less sensitivity towards these sensations (higher physiology‐adjusted thresholds). Conversely, absolute intensity was positively correlated with state anxiety, and less strongly (negatively) correlated with the MAIA not‐distracting. This may suggest individuals with high state anxiety distract themselves from or ignore unpleasant sensations (i.e., low scores on the MAIA not‐distracting subscale), thus requiring a greater interoceptive stimulus (higher absolute intensity). Interestingly, this subscale appears to capture two distinct approaches to navigating unpleasant sensations; attenuation/monitoring and blunting/distraction (Myers and Derekshan 2000), which may contribute to the limited internal consistency of the scale (Mehling 2016) and divergent findings here.

While the proposed physiology‐adjusted measure offers a more robust approach for measuring interoceptive sensitivity, a few methodological limitations should be acknowledged. First, due to data quality issues, the minimum number of participants (72) required to achieve 80% power was not achieved. Thus, all results should be interpreted with caution. Second, the relatively low number of trials limits the precision of the estimated psychometric function. Although the slope and lapse parameters were fixed to allow for reliable estimation of threshold under these constraints, estimating a slope for each individual would increase the accuracy of their threshold estimate and the fit of the individual psychometric function if adequate data allowed. However, the inclusion of an individual slope estimate would require substantially more trials, as over 400 interoceptive trials are required for the estimation of slope (Kingdom and Prins 2010). Importantly, the inclusion of more trials is likely to compromise feasibility due to fatigue and tolerability of the task. The RRST involves breathing through a mouthpiece and adjusting breathing patterns to synchronise with the trial cues, which is not only cognitively and physically tiring, but can also cause mild hyperventilation side effects such as light‐headedness (Meuret and Ritz 2010; Nikolova et al. 2022). Finally, we excluded participants with performance below 70% accuracy, as inconsistent breathing patterns and reduced physiological signal at this level precluded stable convergence of the psychometric function. While this ensured data quality, we recognise that excluding participants with low accuracy may inadvertently omit individuals with genuinely low sensitivity. Although, the proposed method offers an advancement over previous obstruction based metrics by accounting for individual physiological variance, it remains constrained by a minimum ‘signal‐to‐noise’ floor. Future approaches may consider real‐time physiological feedback to help participants maintain the necessary respiratory consistency required for threshold estimation or use an alternative statistical method to adjust for physiological variance (such as a fully Bayesian approach).

Future work should explore using a physiology‐adjusted measure of sensitivity in clinical populations (Paulus 2013; Kato et al. 2018; Soroka et al. 2025), to assess whether physiology‐adjusted metrics offer improved prediction of treatment responses and improved estimation of sensitivity in populations with significantly altered breathing patterns (such as individuals with asthma; Eggart and Valdés‐Stauber 2021).

## 6 Conclusion

Interoceptive dysfunction within anxiety has become a major area of interest and is now a fundamental aspect of many theories related to mental health. However, many methods employed to measure interoceptive dimensions have significant limitations, including the inability to safely perturb homeostatically relevant signals in a controlled manner. Here, we have developed an analysis protocol that captures the inherent physiological variance associated with interoceptive breathing protocols, allowing for more robust measurements of breathing‐related interoceptive sensitivity.

## Funding

This study was supported by Royal Society Te Apārangi (Grant RDF‐UOO1902).

## Conflicts of Interest

The authors declare no conflicts of interest.

## Data Availability

The data that support the findings of this study are available on request from the corresponding author. The data are not publicly available due to privacy or ethical restrictions.
